# The Cell Cycle-Associated Protein CDKN2A May Promotes Colorectal Cancer Cell Metastasis by Inducing Epithelial-Mesenchymal Transition

**DOI:** 10.3389/fonc.2022.834235

**Published:** 2022-03-03

**Authors:** Wei-Kun Shi, Yun-Hao Li, Xue-Shan Bai, Guo-Le Lin

**Affiliations:** ^1^ Department of General Surgery, Peking Union Medical College Hospital, Peking Union Medical College and Chinese Academy of Medical Sciences, Beijing, China; ^2^ Plastic Surgery Hospital, Chinese Academy of Medical Sciences and Peking Union Medical College, Beijing, China

**Keywords:** CDKN2A, colorectal cancer, epithelial-mesenchymal transition, cancer metastasis, cell proliferation and migration

## Abstract

Colorectal cancer (CRC) is a common gastrointestinal malignancy, and recurrence and metastasis contribute considerably to its high mortality. It is well known that the epithelial-mesenchymal transition (EMT) accelerates the rate of cancer cell dissemination and migration, thus promoting cancer metastasis. Targeted therapy is a common modality for cancer treatment, and it can play a role in inhibiting cancer progression. In this study, bioinformatics was used to search for genes associated with the prognosis of CRC. First, differential analysis was performed on colon and rectal cancer samples to obtain 2,840 and 3,177 differentially expressed genes (DEGs), respectively. A Venn diagram was then used to identify 262 overlapping genes from the two groups of DEGs and EMT-related genes. The overlapping genes were subjected to batch survival analysis and batch expression analysis successively, and nine genes were obtained whose high expression in CRC led to a poor prognosis. The least absolute shrinkage and selection operator (LASSO) prognostic model was then constructed to obtain the risk score formula. A nomogram was constructed to seek prognostic independent factors to obtain CDKN2A. Finally, CCK-8 assay, flow cytometry and western blotting assays were performed to analyze the cellular biological function of CDKN2A. The results showed that knockdown of CDKN2A expression inhibited HT-29 cell proliferation, promoted apoptosis and cell cycle progression, and affected the EMT process in CRC.

## Introduction

Colorectal cancer (CRC) is a common gastrointestinal malignancy and the leading cause of cancer deaths. The 2020 global cancer statistics show that there were nearly 1.9 million new cases of CRC (9.8% of cases) and more than 900,000 deaths (9.2% of cases) (1). The five-year survival rate for CRC is highly stage-dependent: early-stage survival rates exceed 90%, while advanced-stage rates reach only 10% (2). This is because early-stage CRC is usually not easily detectable and is not diagnosed until it has spread substantially. Patients with CRC often exhibit symptoms such as dyspareunia, colorectal bleeding, and changes in gastrointestinal motility (3). The causes of the disease are mostly related to poor lifestyle habits, for example, smoking, an unhealthy diet, alcohol abuse, and a lack of physical activity (4). Fortunately, the risk of CRC can be reduced through effective preventive measures, such as cancer screening and a healthy lifestyle (5). However, even though many screening tools can be used to detect and reduce the incidence of CRC, nearly a quarter of cases are still diagnosed with advanced metastases (6). Statistically, the five-year survival rate of patients with metastatic CRC is less than 15%, which is a significant concern (7).

The key characteristic of epithelial-mesenchymal transition (EMT) is the acquisition of a mesenchymal phenotype by epithelial cells (8). In a normal physiological context, EMT promotes developmental processes and wound healing (9). However, in tumor progression, EMT is a dynamic process. Cancer cells adjust their metabolism to meet their growth demands, and certain metabolic pathways involved can directly contribute to EMT (10). In this context, EMT promotes cancer cell dissemination and migration by enhancing cell mobility, which leads to cancer metastasis (11). The hallmark alterations observed in cells undergoing EMT are reduced E-cadherin expression and enhanced N-cadherin and vimentin expression, which usually occur prior to tumor invasion (12). These modifications are mediated by multiple transcription factors (TFs) that directly repress E-cadherin expression and promote the change of epithelial cells to a mesenchymal state (13). In a variety of cancers, including CRC, EMT confers metastatic and stem cell properties on cancer cells (14). There is also evidence that EMT is associated with drug resistance to multiple drugs (15). Therefore, it is important to develop EMT inhibitors for cancer therapy.

Targeted therapies play a unique role in inhibiting cancer progression by directly inhibiting cell proliferation, differentiation, and migration (16). The activity of the TFs involved in EMT can be regulated by a variety of kinases, and their signaling pathways can also serve as effective therapeutic targets. Previous studies have shown that the activation of certain signaling pathways can promote EMT in cancer cells (17). In addition, EMT can be induced by proteins and selectively trigger gene expression programs in cancer stem cells (18).

Therefore, in this study, we focused on identifying EMT-related genes that are potentially associated with CRC prognosis and conducted *in vitro* experiments to validate the biological functions of these potential genes.

## Materials and Methods

### Data Sources

Gene expression matrices and RNA-seq data of colon and rectal cancers were obtained from the TCGA database, as well as mRNA expression data of the corresponding normal tissue samples. The data contained 455 colon cancer samples and 165 rectal cancer samples, and they corresponded to 41 and 10 normal tissue samples, respectively. EMT-related gene sets were downloaded from the Molecular Signatures Database (MsigDB, https://www.gsea-msigdb.org/gsea/msigdb/) and contained 1,263 genes.

### Differential Analysis

The R package Limma was used to study the differential expression of mRNAs, and P < 0.05 and |log2FC| > 1 were defined as the screening threshold for differential expression. The intersection of differentially expressed genes (DEGs) and EMT-related genes was determined using a Venn diagram constructed to obtain overlapping genes associated with colon cancer, rectal cancer, and EMT.

### Construction of the LASSO Prognostic Model

The prognostic feature model was constructed using the R package glmnet to investigate the relationship between genes and CRC prognosis. The least absolute shrinkage and selection operator (LASSO) regression algorithm was used to select features using 10-fold cross-validation. The LASSO prognostic model is a risk score formula that includes multiple genes. The included samples were divided into two groups: high risk and low risk. The log-rank test was used for the survival analysis and to compare the survival difference between the two groups, and timeROC analysis was used to compare the predictive accuracy and risk scores of the pivotal genes.

### Survival Analysis

Gene expression levels in cancer versus para cancer cells were compared using the t-test, and the R package ggplot2 was used for picture plotting. The R package survival was used to analyze survival differences between high- and low-expression groups, and the results are presented as KM survival curves, with P values and hazard ratios (HR) with 95% confidence intervals (95% CI) obtained by log-rank test and univariate Cox proportional hazards regression. Univariate and multivariate Cox analyses were used to identify prognostic independent factors. The R package forest plot was used to draw forest plots, and the R package “rms” was used to create column line plots (nomograms). P value < 0.05 was considered statistically significant.

### Cell Culture

Human HT-29 CRC cells (HTB-38™) were purchased from the American Type Culture Collection (ATCC). The HT-29 cell line was cultured in McCoy’s 5A medium containing 10% FBS in a humidified incubator with 5% CO_2_ at 37°C.

### SiRNA Transfection

siRNA specifically targeting CDKN2A were designed and synthesis by GenePharma Corporation (Shanghai, China), the sequence of sense is GGGUCCCAGUCUGCAGUUATT, the sequence of antisense is UAACUGCAGACUGGGACCCTT. Cells were added to 6-well plates and cultured for 24 h. siRNA was transfected into cells using Lipofectamine 2000 (Thermo Fisher Scientific) according to the manufacturer’s instructions. The medium was replaced after 6 h with fresh medium containing 10% FBS, and the cells were collected after 24 h for the following assays. The negative control group consisted of si-NC transfection-treated HT-29 cells, which were cultured in parallel, as described above.

### Cell Proliferation Assay

Cells were added to 96-well plates and incubated overnight for cell transfection. They continued to be incubated at 37°C in a humid atmosphere of 5% CO_2_. CCK-8 reagent was added to the wells 0, 6, 12, 24, 48, or 72 h post-transfection, and the cells were incubated for 2 h. The absorbance values (optical density, OD) were measured at 450 nm using an enzyme marker.

### Western Blotting

Cells were lysed using RIPA buffer, and the total protein concentration was determined using the BCA assay. Total proteins were separated using 10% SDS-PAGE and transferred to polyvinylidene difluoride membranes. The membranes were blocked with 5% skim milk diluted in TBST for 1 h and then incubated with primary antibodies overnight at 4°C. The primary antibodies were anti-CDKN2A (ab185620, abcam), anti-N-cadherin (ab245117, abcam), anti-E-cadherin (ab40772, abcam), anti-vimentin (ab92547, abcam), and anti-β-actin (ab8226, abcam). After washing, the secondary antibody coupled to horseradish peroxide was added, and the membranes were incubated for 2 h at room temperature. Protein bands were detected using an enhanced chemiluminescence kit.

### Flow Cytometry Assay

The effect of CDKN2A on the apoptosis and cell cycle processes of HT-29 cells was analyzed using flow cytometry. For apoptosis, the transfected cells were collected and treated with trypsin, and the supernatant was removed after centrifugation at 3,000 × g at room temperature. Cells were suspended in the binding buffer according to the supplier’s instructions and stained with membrane-linked protein V-FITC/Propidium iodide (PI) in the dark, and then the apoptosis rate was determined using flow cytometry. Similarly, transfected HT-29 cells were collected, fixed overnight in 75% ethanol at 4°C, stained with PI according to the manufacturer’s instructions, and subsequently subjected to a cell cycle assay using flow cytometry.

## Results

### Differential Analysis

**Figure 1:**
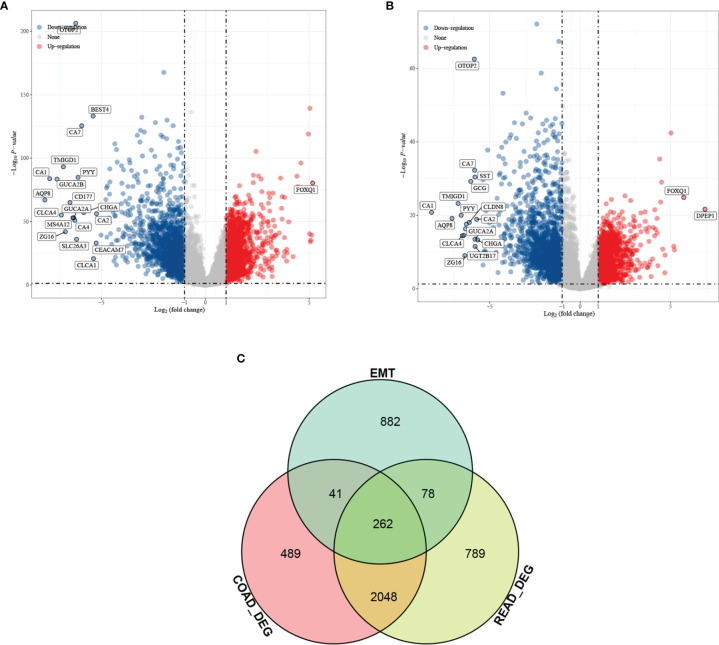
Differential analysis results from screening of EMT-related genes associated with prognosis of colorectal cancer (CRC). Volcano plot. Red indicates upregulated genes, and blue indicates downregulated genes. **(A)** Differential analysis of colon cancer and para cancer; **(B)** differential analysis of rectal cancer and para cancer; and **(C)** intersection results of differentially expressed genes (DEGs) in colon cancer, DEGs in rectal cancer, and EMT-related genes.

**Figure 2:**
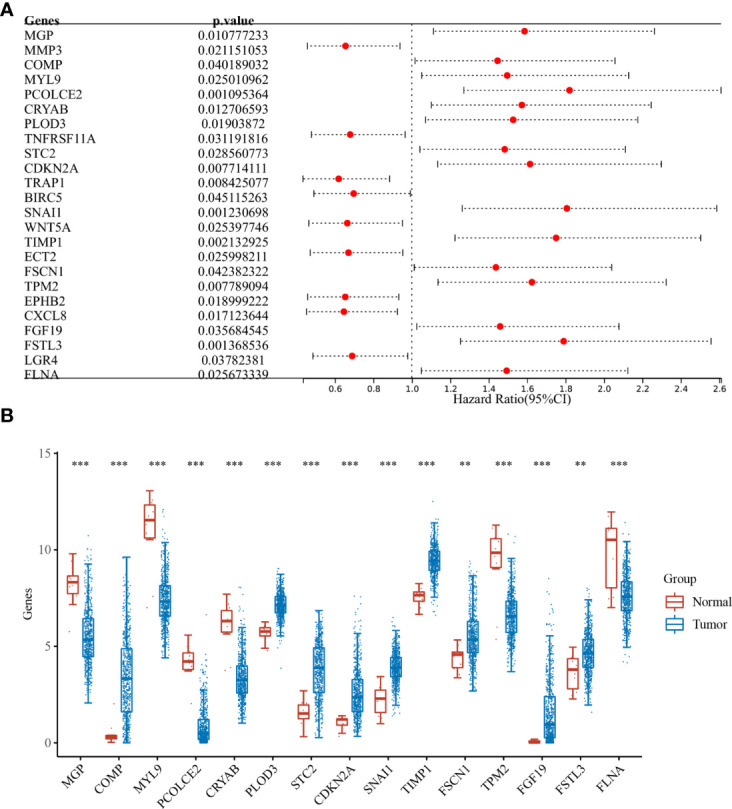
Batch survival and expression analysis. **(A)** Results of batch survival analysis of overlapping genes, HR > 1 indicates that the gene is a protective factor for cancer; and **(B)** expression analysis of genes with survival significance. **P < 0.01, ***P < 0.001.

Differential analysis was performed for colon cancer (T = 455, Nor = 41) and rectal cancer (T = 165, Nor = 10), and the results showed that there were 1,401 upregulated genes and 1,439 downregulated genes in colon cancer (**Figure 1A**), and 1,356 upregulated genes and 1,821 downregulated genes in rectal cancer (**Figure 1B**). The intersection results showed that there were 262 overlapping genes among the differential genes and EMT-related genes (**Figure 1C**). Survival analysis was performed on the 262 overlapping genes, of which 24 genes were associated with prognosis (**Figure 2A**). The genes with a risk ratio (HR) > 1 were subsequently subjected to bulk expression analysis, and nine genes were highly expressed in CRC tissues and six genes were highly expressed in paracancer tissues (**Figure 2B**).

### Construction of the Prognostic Risk Model

**Figure 3:**
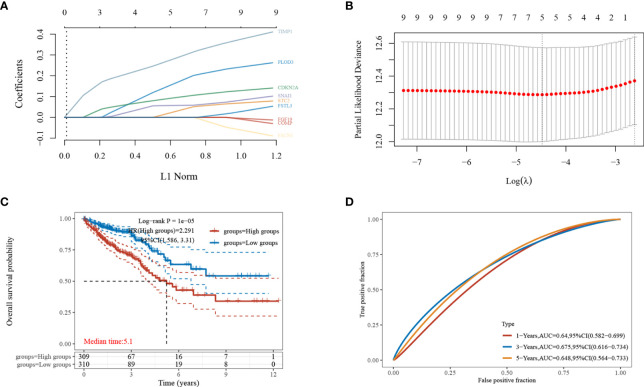
Establishment of the least absolute shrinkage and selection operator (LASSO)-Cox prognostic model. **(A, B)** Selected characteristic coefficients are shown by lambda parameters, and the number of prognostic factors was determined using LASSO regression analysis; **(C)** distribution of high and low risk samples;**(D)** KM survival analysis of the high- and low-risk samples for CRC in the TCGA dataset.

The nine EMT-related genes that were found to be highly expressed in CRC cells were subjected to LASSO regression analysis to construct characteristic prognostic models (**Figures 3A, B**). When the minimum lambda value was 0.0114, the following risk score formula was obtained: Riskscore = (0.1879)*PLOD3+(0.0427)*STC2+(0.1019)*CDKN2A+(0.0578)*SNAI1+(0.3033)*TIMP1. According to the calculation results of the formula, the samples are divided into high-risk group and low-risk group (**Figure 3C**). The KM survival curves demonstrated the difference in survival between the high-risk and low-risk groups, with the low-risk group found to have better survival compared to the high-risk group (**Figure 3D**).

### CDKN2A Is an Independent Prognostic Factor for Colorectal Cancer (CRC)

**Figure 4:**
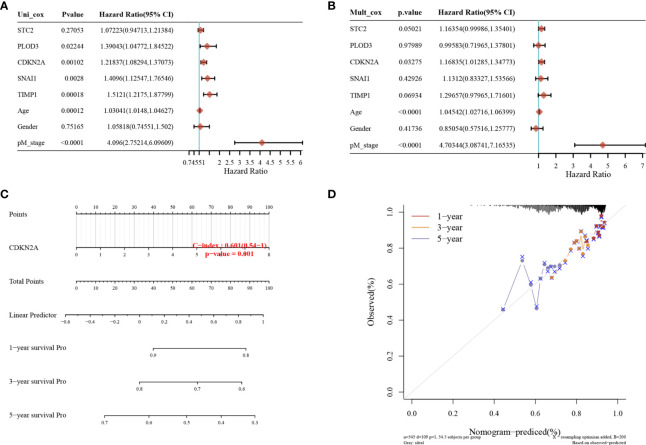
Identification of independent prognostic factors for CRC and single-gene prognostic analysis. **(A)** Single-factor Cox regression analysis; **(B)** multi-factor Cox regression analysis; and **(C, D)** the nomogram model used to predict the OS of patients with CRC at one, three, and five years. p < 0.05 was considered statistically significant.

Genes in the risk score formula were defined as risk genes, and a nomogram was constructed to analyze the prognostic value of the five risk genes. Combining the results of the univariate and multifactorial Cox analyses showed CDKN2A to be an independent prognostic factor for CRC, and that age and M stage are also significant (**Figures 4A, B**). The nomogram model had good predictive power and demonstrated the prognostic predictive ability of each factor for CRC (**Figures 4C, D**).

### Comparison of CDKN2A Expression With Survival

**Figure 5:**
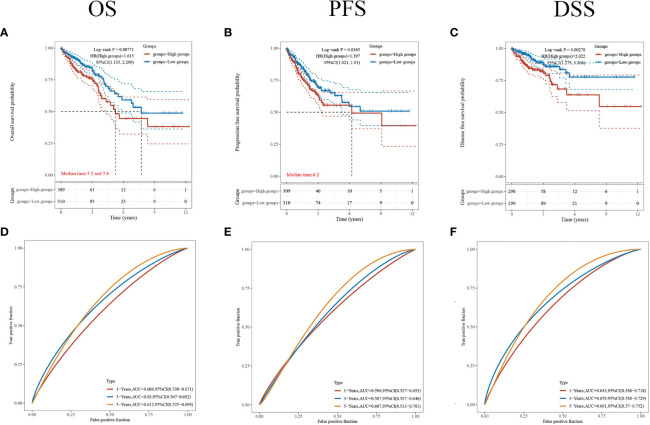
Comparison of different survival types. **(A–C)** The OS, PFS and DSS of patients with CRC who expressed high levels of CDKN2A. **(D–F)** ROC curves of OS, PFS and DSS. p < 0.05 was considered statistically significant.

To further understand the prognostic significance of CDKN2A in CRC, we analyzed the prognosis in terms of the three survival types. The results for overall survival (OS) (**Figure 5A**), progression-free survival (PFS) (**Figure 5B**), and disease-specific survival (DSS) (**Figure 5C**) showed that high CDKN2A expression indicated a worse prognosis. The median OS time was 28.8 months longer for patients with low CDKN2A expression compared to those with high CDKN2A expression. The ROC curves of OS (**Figure 5D**), PFS (**Figure 5E**) and DSS (**Figure 5F**) show the area under the curve (AUC) for this risk score is around 0.6.

### Knockdown of CDKN2A Expression Inhibits HT-29 Cell Proliferation

**Figure 6:**
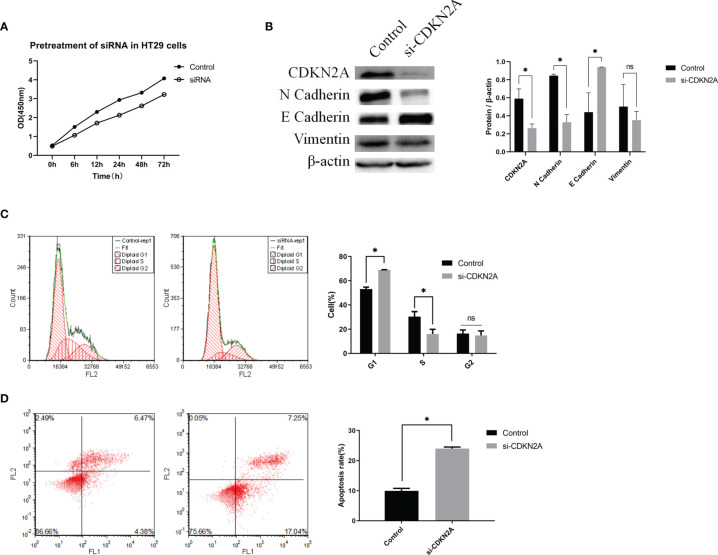
*In vitro* experiments validating the biological role of CDKN2A. **(A)** CCK-8 assay; **(B)** Protein expression of CDKN2A, E-cadherin, N-cadherin, and vimentin. **(C)** Flow cytometric analysis of the cell cycle and statistical results of the cell cycle; **(D)** Flow cytometric analysis of apoptosis and statistical results of apoptosis; The results are shown as the mean ± SD of the data from three sets of replicate experiments, *P < 0.05, ns, non significant.

The cell proliferative ability was then determined *via* a CCK-8 assay. The results showed that knockdown of CDKN2A expression inhibited cell proliferation (**Figure 6A**).

### CDKN2A Promotes CRC Progression *Via* Epithelial-Mesenchymal Transition (EMT)

To verify the relationship between CDKN2A and EMT in CRC cells, we determined the protein levels of EMT signaling pathway-related molecules using WB. The results showed that knockdown of CDKN2A expression in HT-29 cells led to enhanced of E-cadherin expression and suppression expression of N-cadherin and vimentin at the protein levels compared with the negative control cells (**Figure 6B**). Thus, it is evident that knockdown of CDKN2A expression inhibits EMT in CRC cells.

### CDKN2A Is Involved in the Regulation of Cell Cycle and Apoptosis of HT-29 Cells

Flow cytometry was utilized to understand the effect of CDKN2A on the cell cycle and apoptosis. Compared with the negative control cell samples, the percentage of G1 phase cells in the treated HT-29 cell samples increased significantly (**Figure 6C**). Also, the percentage of apoptotic HT-29 cells was significantly higher in the cell samples with knocked-down CDKN2A than in the control cell samples (**Figure 6D**).

## Discussion

CRC is a highly lethal cancer, and its mortality is mainly attributed to recurrence and distant metastasis (19). There is a strong association between EMT and tumor metastasis, and EMT has been identified as a major cause of CRC metastasis (20). In this study, bioinformatics analysis showed that CDKN2A(p14) was an independent prognostic factor of colorectal cancer, and its high expression could induce EMT and mediate adverse clinical outcomes in CRC patients.

CDKN2A mainly encodes two proteins, p14 and p16 (21). p16 protein is a cyclin dependent kinase inhibitor, which can bind with CDK4 and CDK6 and prevent the phosphorylation of retinoblastoma protein, so as to prevent the process of cell cycle (22). p14 protein is a splice variant of CDKN2A. It has no amino acid homology with p16 due to frameshift, and can regulate the activity of p53 (23). As a tumor suppressor, CDKN2A is involved in B-cell differentiation, cell survival, and cell cycle progression (24). CDKN2A is also associated with cancer prognosis. Deletion of CDKN2A is associated with a poor prognosis in soft tissue sarcomas and is an independent prognostic factor in HPV-negative head and neck squamous cell carcinomas (21, 25). Some reports suggest that hypermethylated CDKN2A is a predictor of poor prognosis of colorectal cancer (26). Interestingly, we also found that high CDKN2A expression is an independent prognostic factor for CRC and is associated with a poor prognosis. The reason for the contradiction between the two conclusions may be related to the heterogeneity between samples. In addition, there are regional differences in the risk of poor prognosis of colon cancer caused by CDKN2A methylation. In addition, the mechanism by which CDKN2A promotes CRC progression has been investigated. It may promote the proliferation and metastasis of tumor cells through ILF3-AS1/EZH2/H3K27me3/CDKN2A axis (27). Different from that study, in this study we analyzed the relationship between CDKN2A and colorectal cancer metastasis from EMT.

EMT plays an important role in tumor metastasis. The onset of the EMT process was often accompanied by decreased expression of E-cadherin and enhanced expression of N-cadherin and vimentin. E-cadherin is a transmembrane protein, and its downregulation is usually associated with the invasion of early tumor cells. N-cadherin is commonly found in non-epithelial cells, and its upregulation induces EMT and cancer stem cell properties (28). Vimentin is an intermediate filament protein that regulates cellular traits and migratory capacity during cell metastasis to support the EMT phenomenon (29). It is reported that EMT related prognostic features can not only be used as a prediction tool for high recurrence risk of cancer (30), but also promote invasion and metastasis (31). In the present study, WB results showed that knocking down CDKN2A expression in HT-29 cells was followed by enhanced E-cadherin expression and suppression of N-cadherin and vimentin expression. This implies that CDKN2A can induce EMT. Coincidentally, the occurrence of cancer invasion and metastasis is often related to EMT. In pancreatic cancer and melanoma, CDKN2A is often noticed due to mutations (22, 32). It is also reported that CDKN2A is lost in locally advanced or metastatic tumor tissues (33). Studies have shown that the mutation or defect of CDKN2A in cancer cells can induce EMT, so as to promote the invasion and metastasis of cancer cells (34, 35). The inactivation of p14 protein can worsen colorectal cancer and lead to tumor size (36).

In conclusion, this study combined bioinformatics analysis with *in vitro* assays to identify CDKN2A as an independent prognostic factor in CRC and to confirm its biological role. The results show that CDKN2A (p14) has a protumor effect, and the increase of its expression can induce the occurrence of EMT.

## Data Availability Statement

Publicly available datasets were analyzed in this study. This data can be found here: https://www.gsea-msigdb.org/gsea/msigdb/.

## Author Contributions

WS and YL contributed equally to this work. All authors contributed to the article and approved the submitted version.

## Funding

This work was supported by Beijing Major Science and Technology Projects under grant No. D171100002617003.

## Conflict of Interest

The authors declare that the research was conducted in the absence of any commercial or financial relationships that could be construed as a potential conflict of interest.

## Publisher’s Note

All claims expressed in this article are solely those of the authors and do not necessarily represent those of their affiliated organizations, or those of the publisher, the editors and the reviewers. Any product that may be evaluated in this article, or claim that may be made by its manufacturer, is not guaranteed or endorsed by the publisher.
